# Supplementary material for: Controlling factor of incoming plate hydration at the north-western Pacific margin
Source: Nat Commun. 2018 Sep 21;9:3844. doi: 10.1038/s41467-018-06320-z (PMC6155115; doi:10.1038/s41467-018-06320-z)
Supplement: Supplementary file 1 — Supplementary Information [file 41467_2018_6320_MOESM1_ESM.pdf]

Supplementary information of

Controlling factor of incoming plate hydration at the north-western Pacific margin

Fujie *et al.*

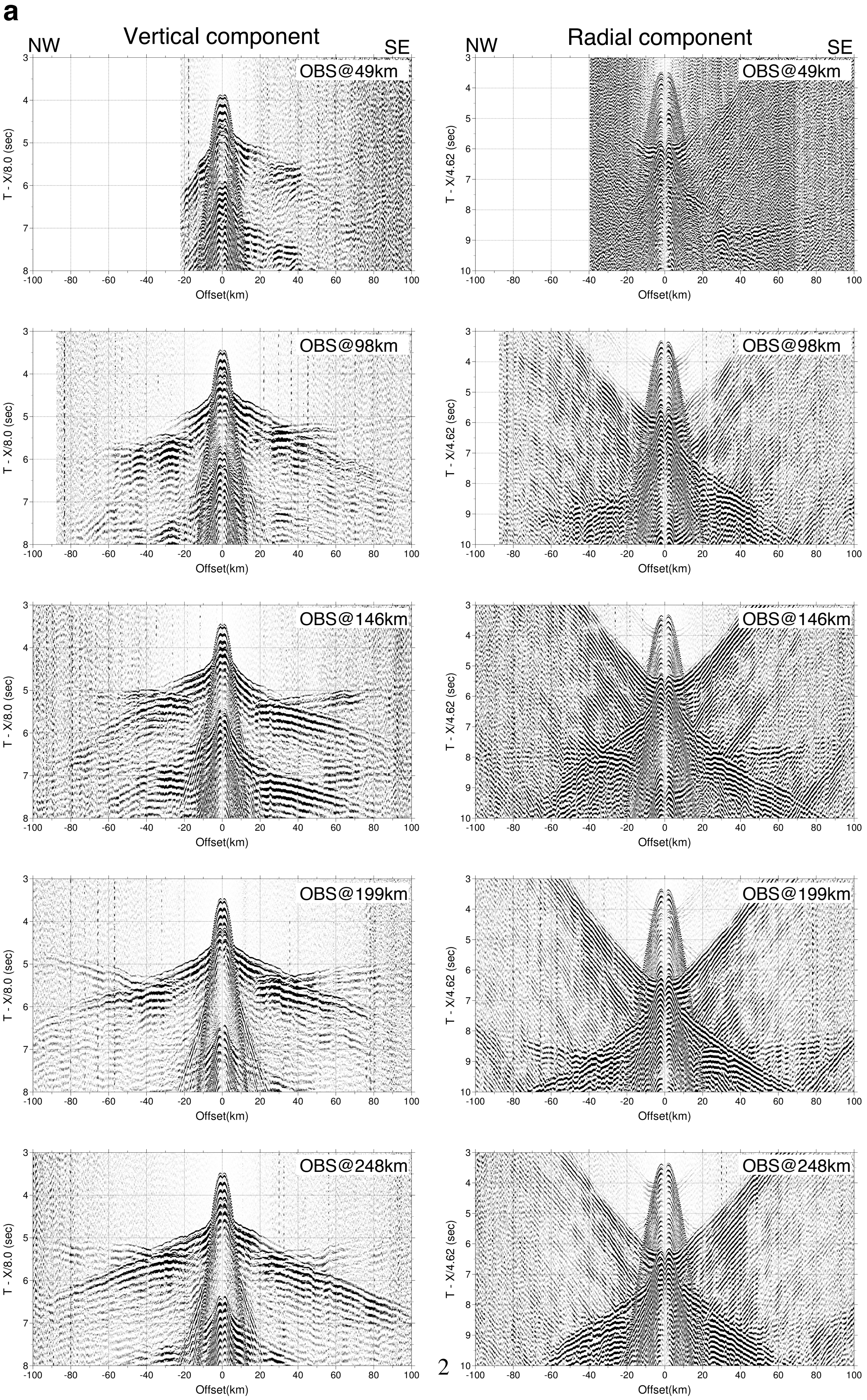
a
Vertical component
Radial component
NW
SE
OBS@49km
OBS@98km
OBS@146km
OBS@199km
OBS@248km
T - X/8.0 (sec)
T - X/4.62 (sec)
Offset(km)

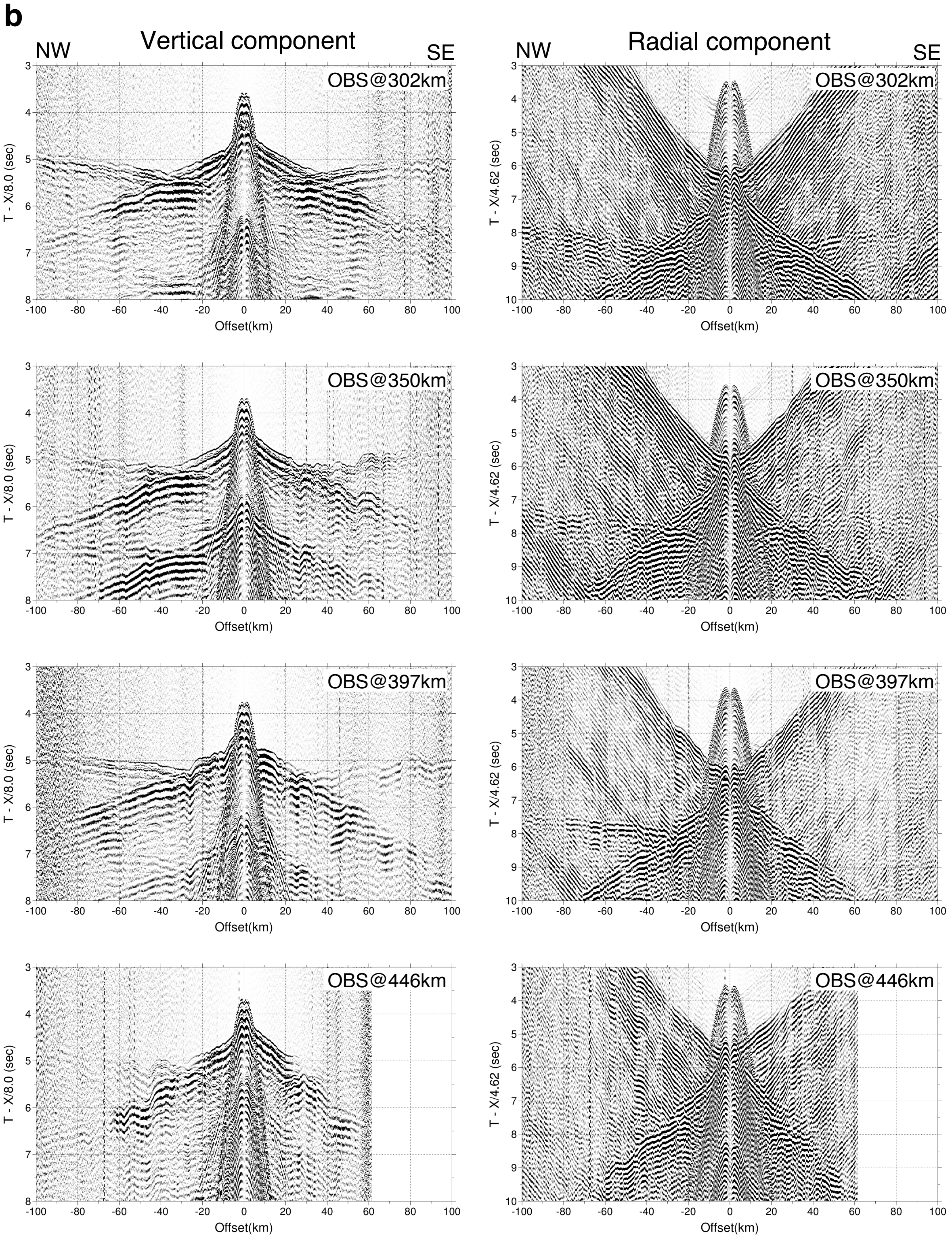

Supplementary Figure 1: OBS sections along the line A2 (Kuril Trench).
(a) Examples of OBS data recorded along the northern half of line A2 (Kuril Trench). Each OBS position is measured from the trench axis, and the bottom axis shows the offset distance between the OBS and air-gun shots. The left column shows vertical component data reduced by 8 km/s after applying a 4-20 Hz band-pass filter. The right column shows radial component data reduced by 4.62 km/s after applying a 4-20 Hz band-pass filter. We calculated the radial

component data from the two orthogonal horizontal components by polarization of the direct water arrivals of the airgun shots. (b) The same as (a) but along the southern half of line A2 (Kuril Trench).

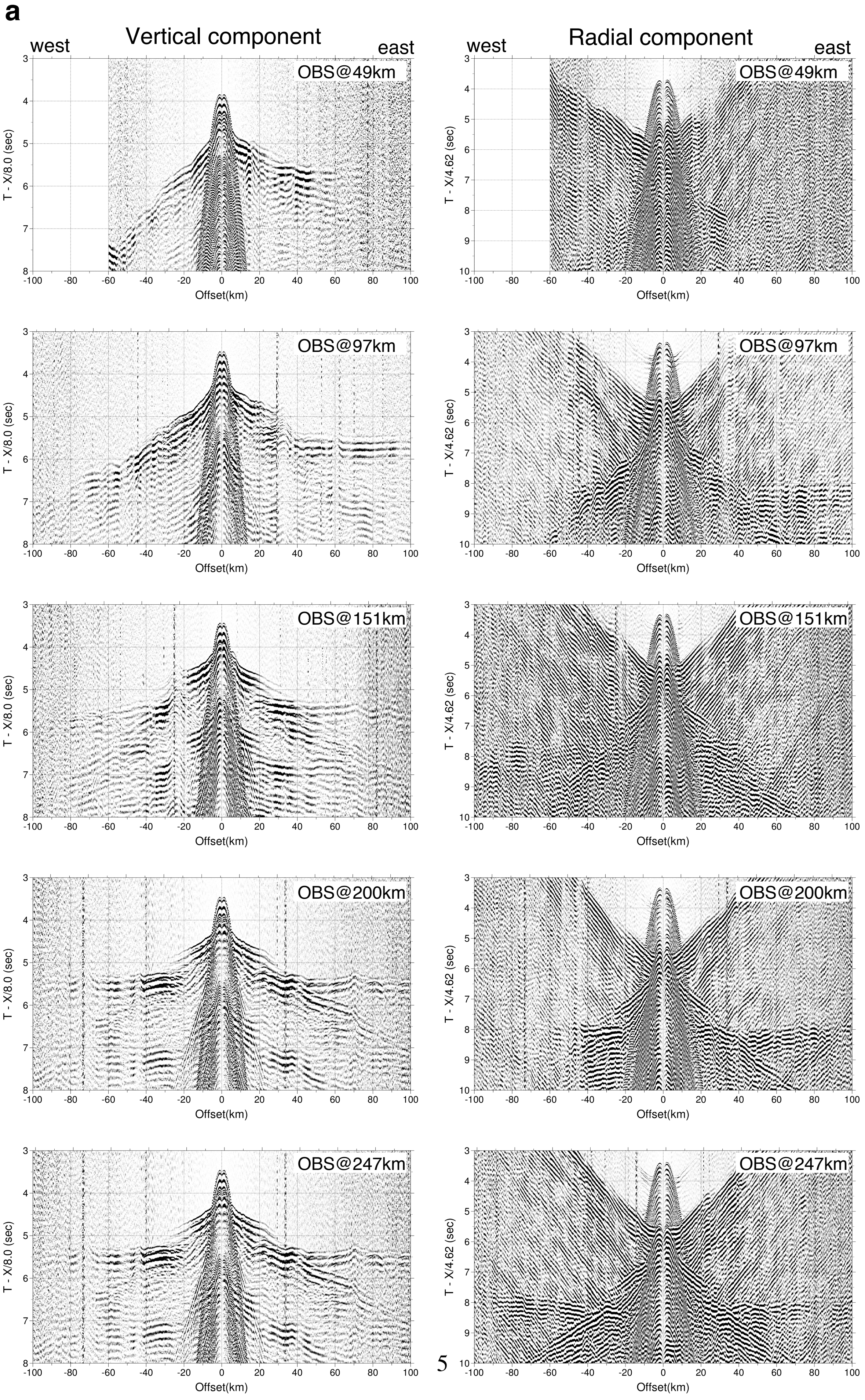
a
Vertical component
Radial component
west
east
OBS@49km
OBS@97km
OBS@151km
OBS@200km
OBS@247km
T - X/8.0 (sec)
T - X/4.62 (sec)
Offset(km)

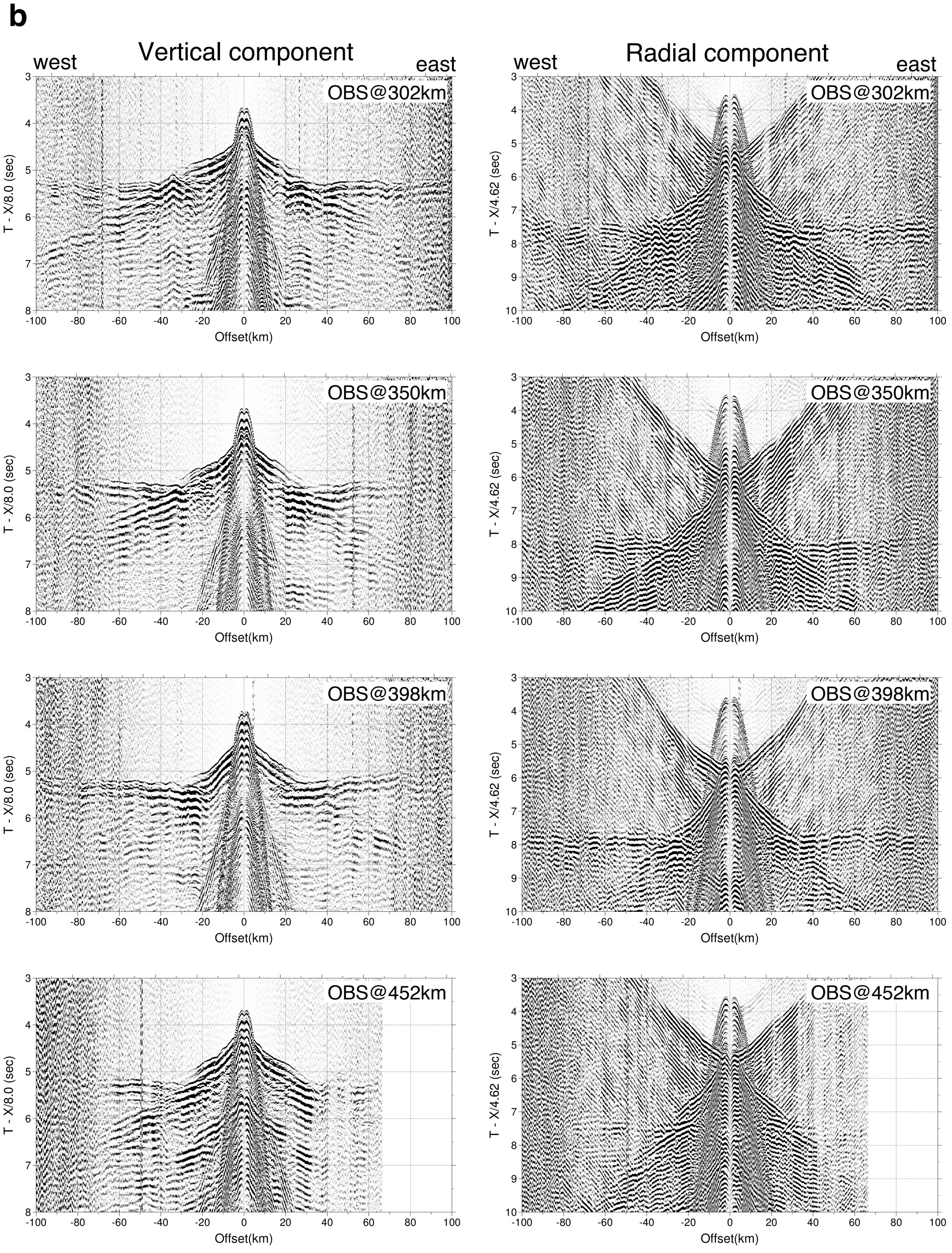

Supplementary Figure 2: OBS sections along the line A3 (Japan Trench).
(a) The same as Supplementary Fig. 1, but along the western half of line A3 (Japan Trench).
(b) The same as Supplementary Fig. 1, but along the eastern half of line A3 (Japan Trench).

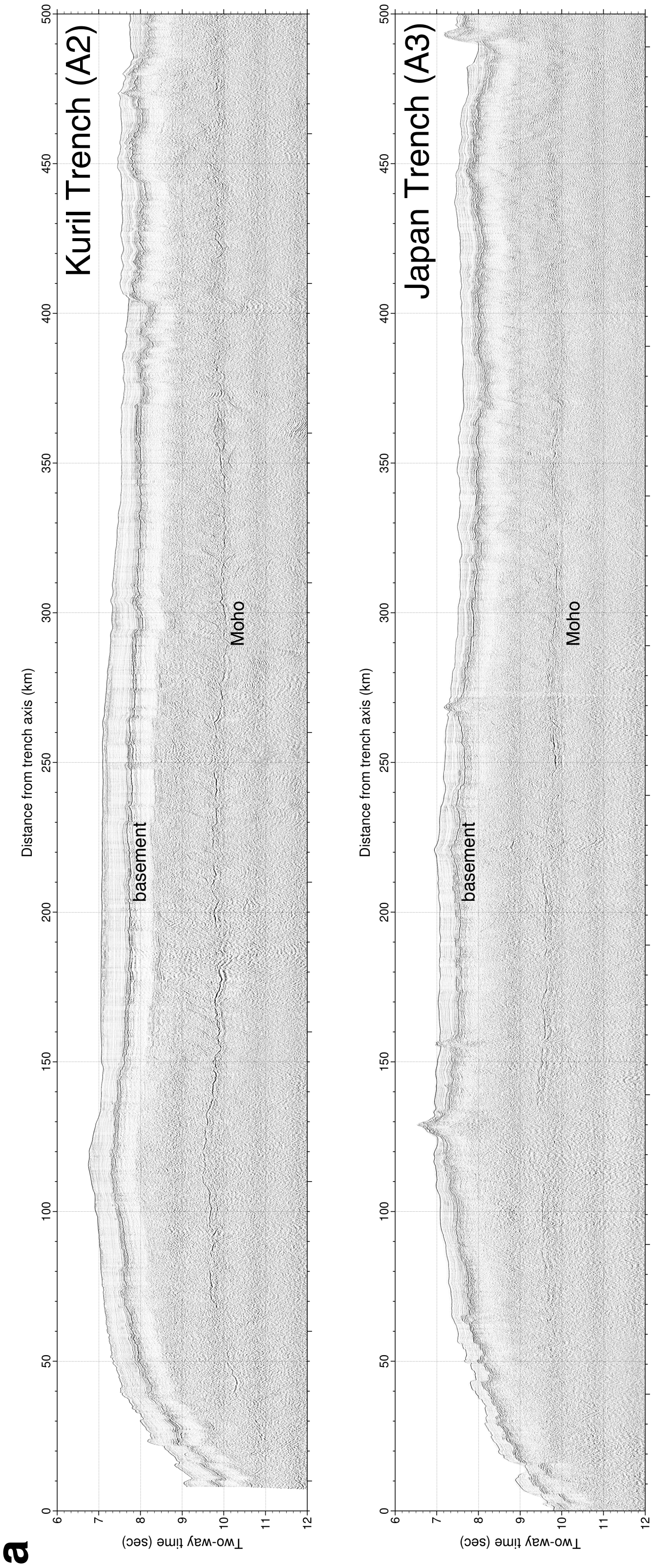
a
Kuril Trench (A2)
Japan Trench (A3)
Distance from trench axis (km)
Two-way time (sec)
basement
Moho

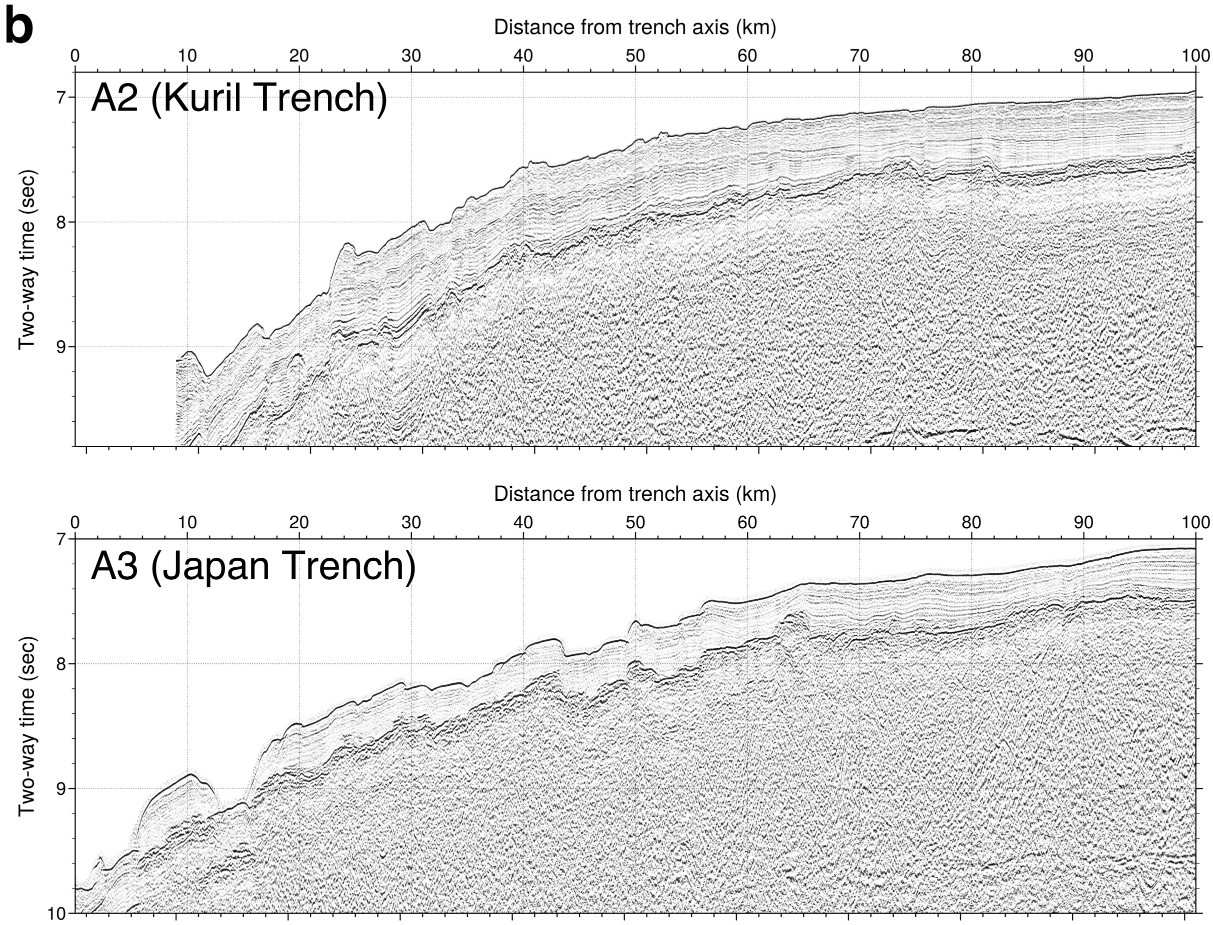
b
Distance from trench axis (km)
0
10
20
30
40
50
60
70
80
90
100
A2 (Kuril Trench)
Two-way time (sec)
7
8
9
Distance from trench axis (km)
0
10
20
30
40
50
60
70
80
90
100
A3 (Japan Trench)
Two-way time (sec)
7
8
9
10

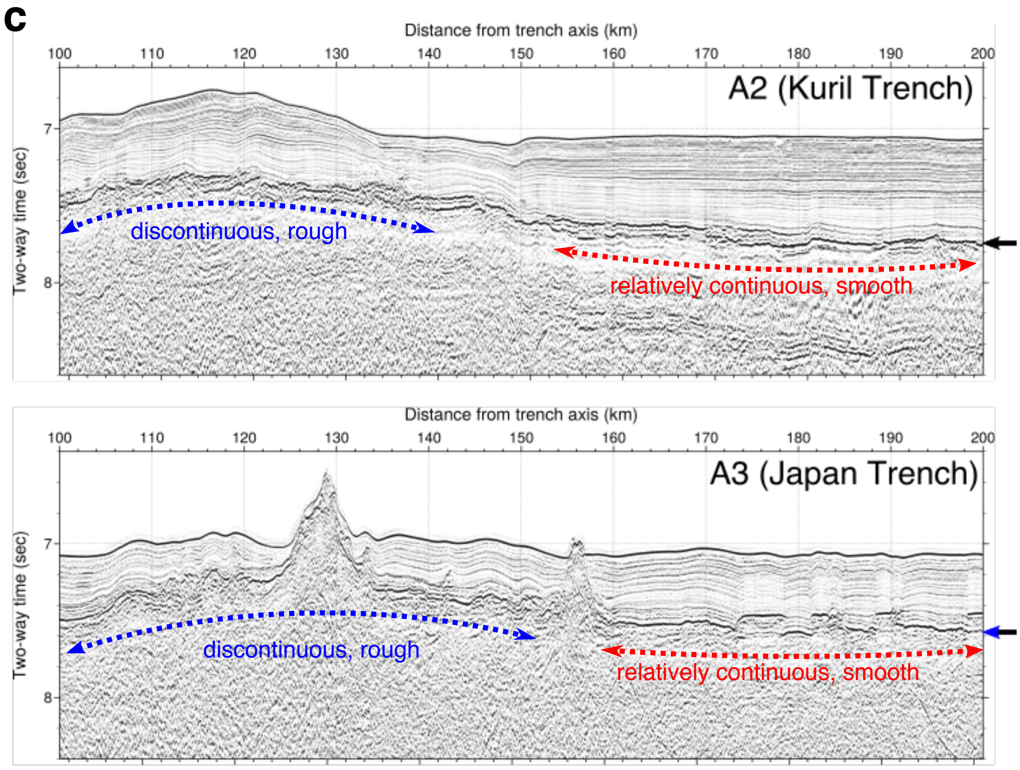

Supplementary Figure 3: Time-migrated seismic reflection sections.
(a) Time-migrated seismic reflection sections along line A2 (Kuril Trench) and line A3 (Japan Trench). Top axes show the distance from the trench axis. The strong reflector just below the seafloor represents the sediment basement. The Moho is located about 2 s below the basement. In the traveltime inversion, we utilized two-way reflection traveltimes from the basement and the Moho as well as wide-angle reflection and refraction traveltimes observed in OBS data. (b) Enlarged time-migrated seismic reflection sections near the trench axis. We can see seafloor topographic fluctuations associated with normal faulting starts to develop at around 80 km from the trench axis. (c) Enlarged time-migrated seismic reflection sections around 150 km from the trench axis. The continuity of the basement reflection (arrows) changes at around 150 km from the trench axis, suggesting the fracturing of the basement starts at around 150 km from the trench axis (Fujie *et al.*, 2013).

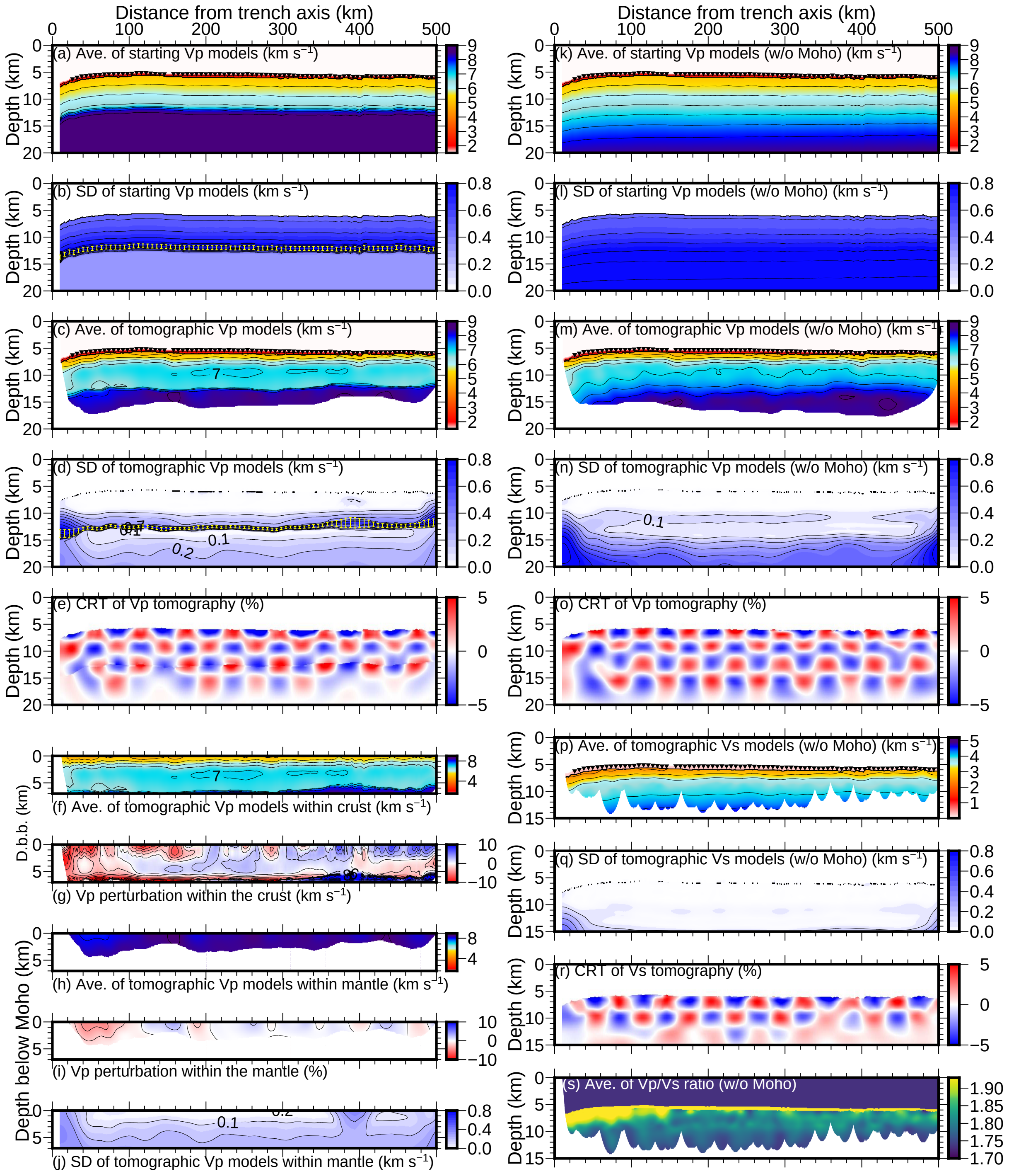

Supplementary Figure 4: Seismic velocity analysis along line A2 (Kuril Trench). The left column shows the results of velocity analysis with 4-layer model parameterization and the right column shows the results with 3-layer model parameterization. (a) Average of 400 starting V_p models and (b) their standard deviations with 4-layer model parameterization. (c) Average of 400 final V_p models after tomographic traveltime inversion and (d) their standard deviations. (e) Result of checkerboard resolution tests (CRT) on one of representative velocity model. (f) Average of 400 final V_p models below the basement and (g) their perturbations relative to the average V_p model. The vertical axis, D.b.b., shows depth below the basement. (h) Average of 400 final V_p models below the Moho, (i) their perturbations

relative to the average V_p model, and (j) their standard deviations. The vertical axis, D.b.M., shows depth below the Moho. (k) Average of 400 starting V_p models and (l) their standard deviations with 3-layer model parameterization. (m) Averages of 400 final V_p models after tomographic traveltime inversion and (n) their standard deviations. (o) Result of CRT on one of representative velocity model. (p) Averages of 400 final V_s models after tomographic traveltime inversion and (q) their standard deviations. (r) Result of CRT on one of representative V_s model. (s) V_p/V_s ratio model calculated from (m) and (p). Areas without rays are white in resultant seismic velocity models and CRT.

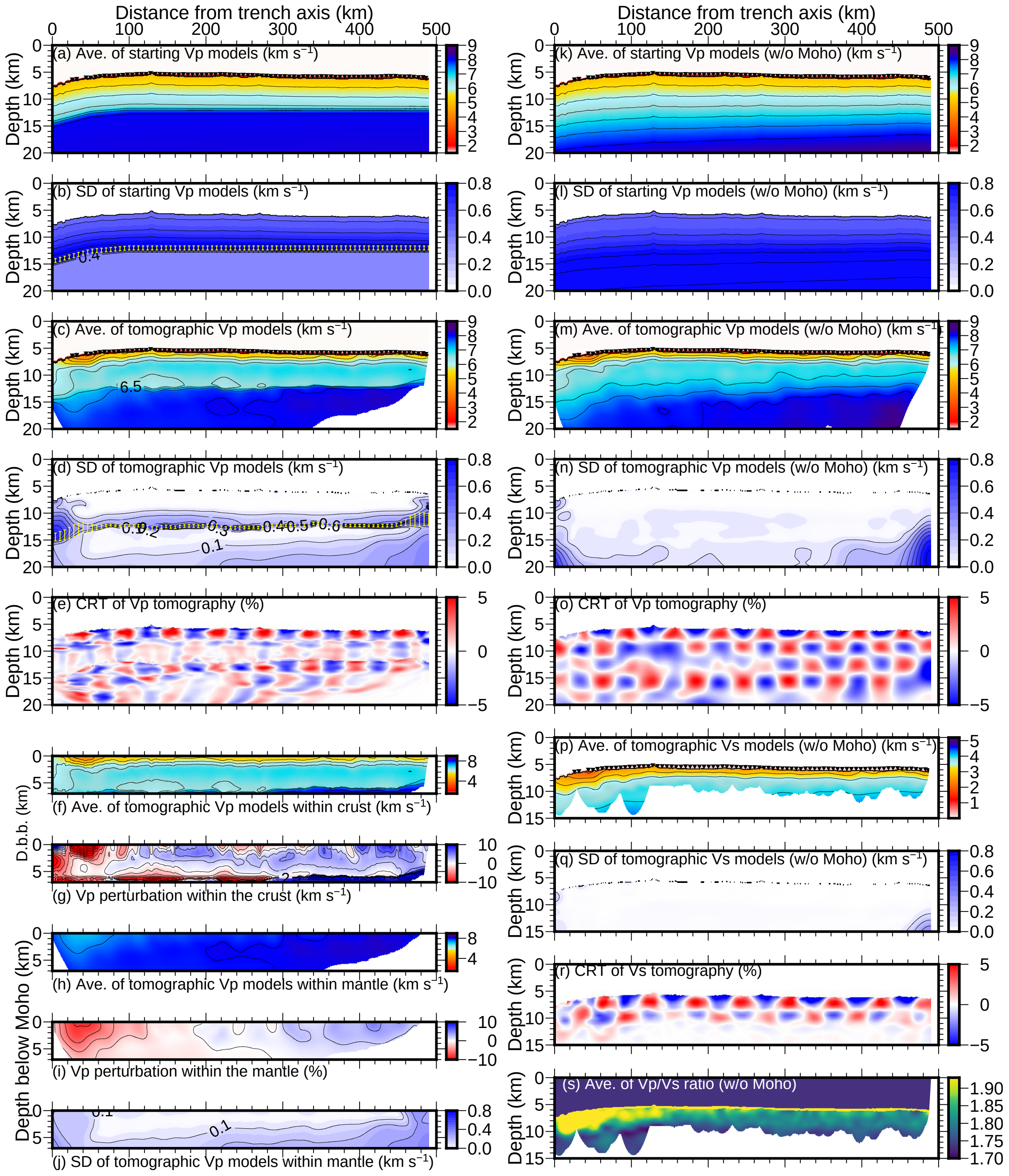

Supplementary Figure 5: Seismic velocity analysis along line A3 (Japan Trench, except OBSs near the trench axis).
The same as Supplementary Fig. 4, but along line A3 (Japan Trench). OBSs deployed in the vicinity of the trench axis (white circles in Fig. 1) were not used to construct these models so that the results could be directly compared with those for the Kuril Trench (Supplementary Fig. 4).

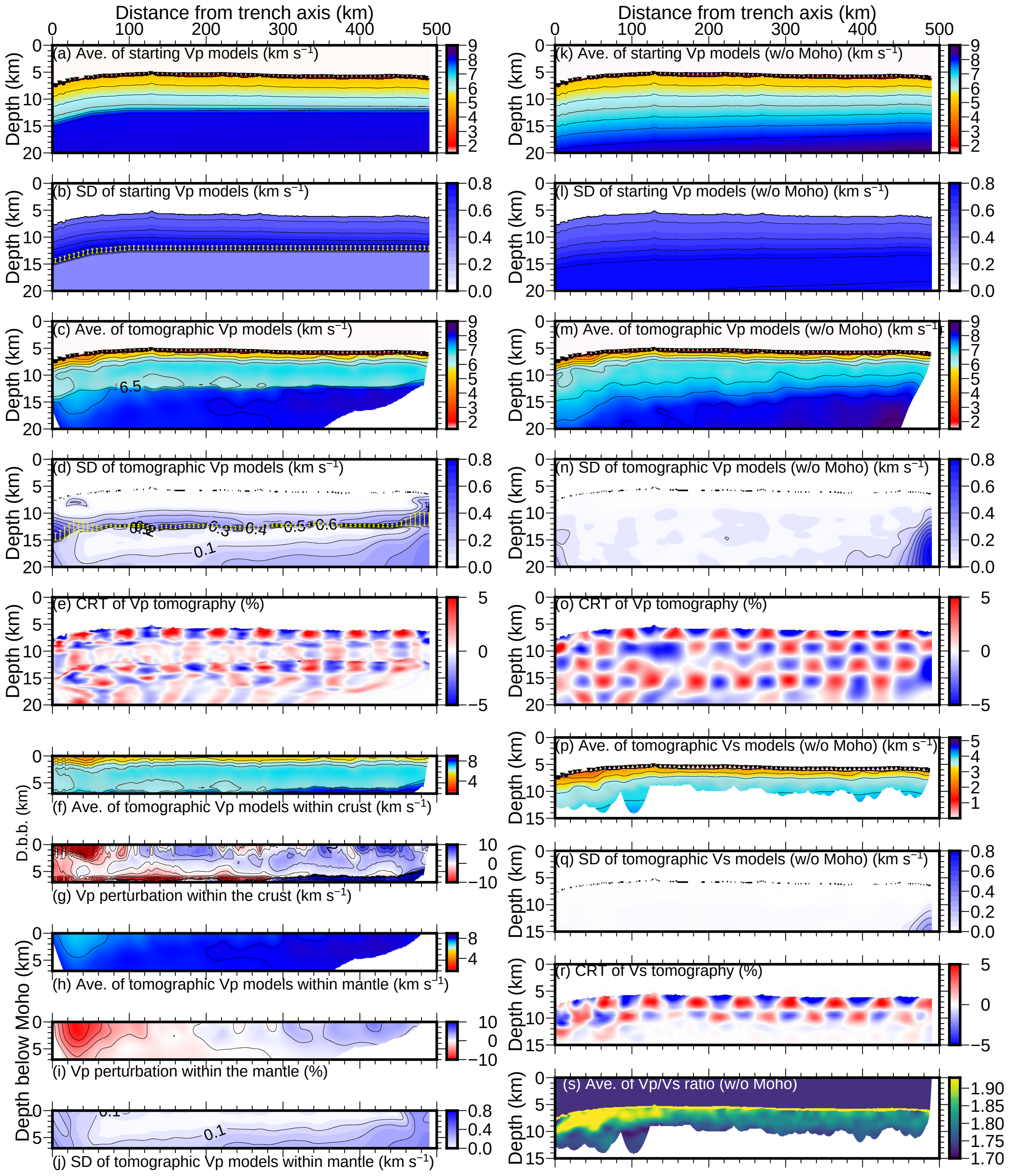

Supplementary Figure 6

Supplementary Figure 6: Seismic velocity analysis along line A3 (Japan Trench, all OBSs).

The same as Supplementary Fig. 5, but all OBSs (shown by green and white OBSs in Fig. 1) were used to construct these models. The differences between Supplementary Fig. 5 and Supplementary Fig. 6 are not significant except at the immediate vicinity of the trench axis.

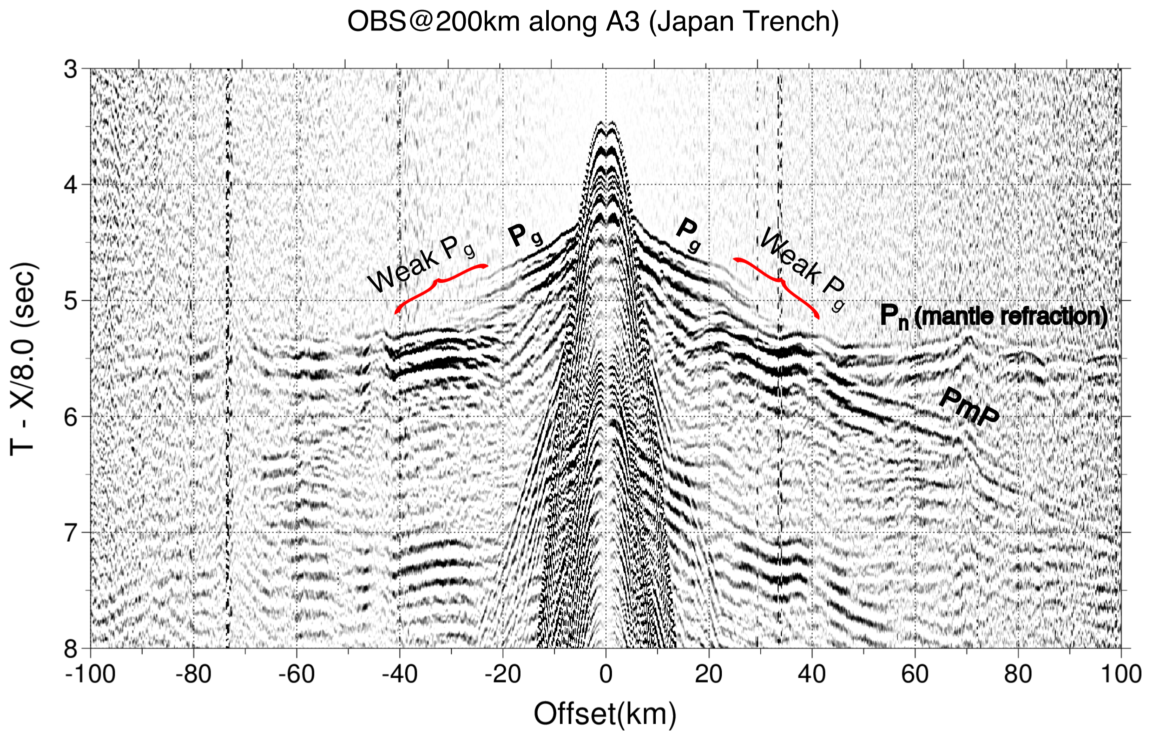

Supplementary Figure 7: OBS data suggesting velocity reversal within the crust. An example record section showing amplitude variations of oceanic crustal refraction (Pg). The Pg becomes weak at the offset distance larger than about 20 km (i.e., shadow zone), suggesting a velocity reversal within the oceanic crust. Obtained V_p models, especially of the Japan Trench, show clear velocity reversal within the oceanic crust at some parts of the survey lines (Fig. 3, Supplementary Figs. 4-S), and distribution of OBSs suggesting a velocity reversal within the oceanic crust is consistent with the modelled V_p. Therefore, the velocity reversal within the crust is considered to be a real feature. However, the velocity reversal is observed regardless of distance from the trench axis and it was not considered to be related with the bend faulting (e.g., Fujie *et al.*, 2016).

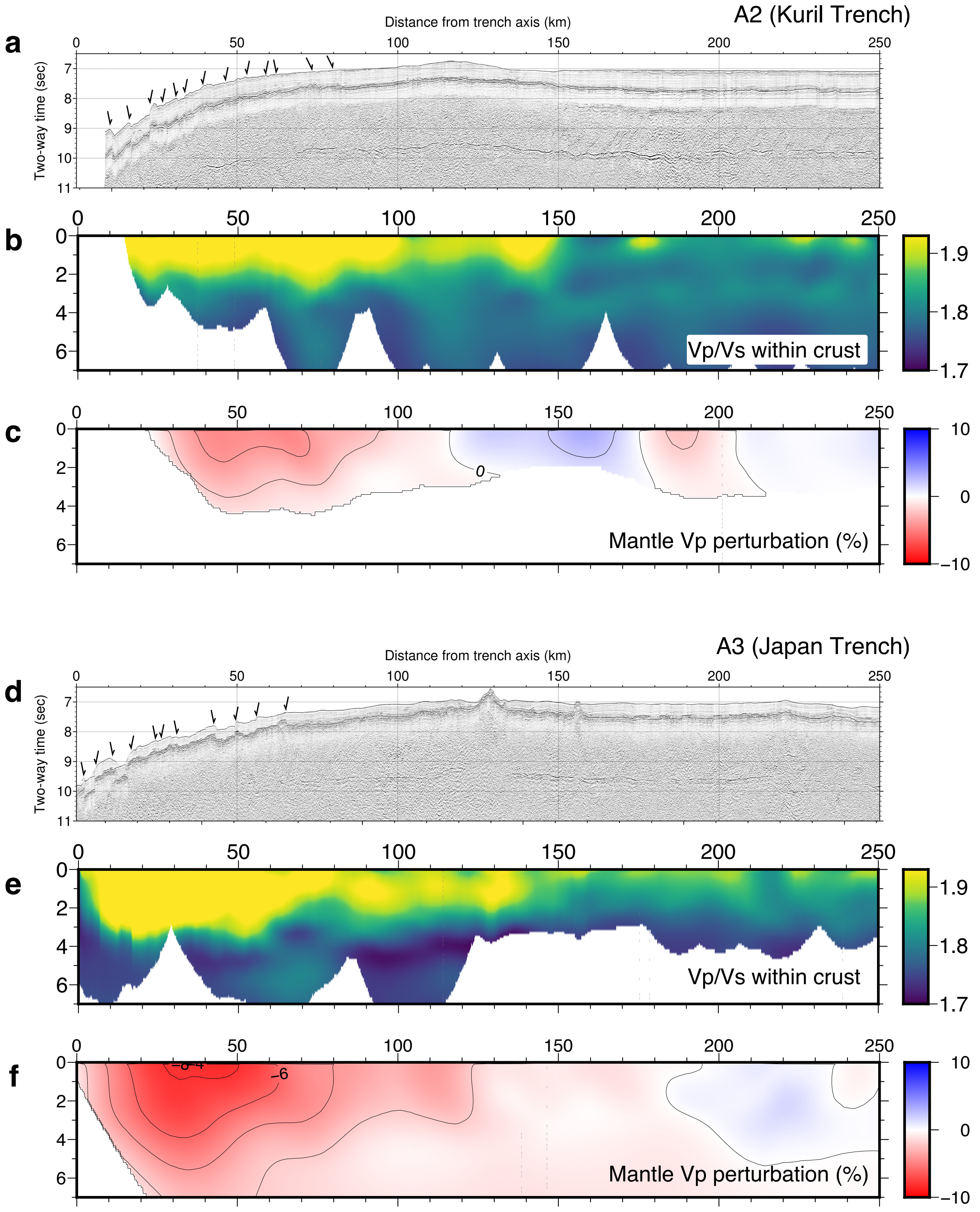

Supplementary Figure 8: Bend faulting and changes in seismic velocities. Comparison of bend faulting and seismic velocity changes between the Kuril Trench and the Japan Trench. V_p/V_s ratio within the crust begins to increase from around 150 km from the trench, where continuity of the basement reflection changes (Supplementary Fig. 3c). Based on these correlation between V_p/V_s ratio and basement reflections, a former study in the Kuril Trench (Fujie *et al.*, 2013) suggested that north of 150 km well-developed fractures enable

water to percolate from the bottom of the sediment into the top of the oceanic crust. In addition, reduction of mantle V_p and increase of the crustal V_p/V_s ratio become more significant near the trench axis where well-developed bend faults are observed at the seafloor. Considering spatial resolution of seismic velocity models, it is fairly certain that these seismic velocity changes are closely associated with the development of plate bending-related fracturing and normal faulting. Note that the magnitude of changes are more remarkable in the Japan Trench than those in the Kuril Trench although changes in seismic velocities begin at similar distance from trench axis.
